# An update on the biological characteristics and functions of tuft cells in the gut

**DOI:** 10.3389/fcell.2022.1102978

**Published:** 2023-01-10

**Authors:** Yixuan Du, Han Gao, Chengwei He, Shuzi Xin, Boya Wang, Sitian Zhang, Fengrong Gong, Xinyi Yu, Luming Pan, Fanglin Sun, Wen Wang, Jingdong Xu

**Affiliations:** ^1^ Department of Oral Medicine, School of Basic Medical Sciences, Capital Medical University, Beijing, China; ^2^ Department of Physiology and Pathophysiology, School of Basic Medical Sciences, Capital Medical University, Beijing, China; ^3^ Undergraduate Student of 2018 Eight Program of Clinical Medicine, Peking University People’s Hospital, Beijing, China; ^4^ Department of Clinical Medicine, School of Basic Medical Sciences, Capital Medical University, Beijing, China; ^5^ Department of Laboratory Animal Research, Xuan Wu Hospital, Capital Medical University, Beijing, China

**Keywords:** tuft cell, intestinal epithelial cell, type 2 immune response, helminth infection, tumor

## Abstract

The intestine is a powerful digestive system and one of the most sophisticated immunological organs. Evidence shows that tuft cells (TCs), a kind of epithelial cell with distinct morphological characteristics, play a significant role in various physiological processes. TCs can be broadly categorized into different subtypes depending on different molecular criteria. In this review, we discuss its biological properties and role in maintaining homeostasis in the gastrointestinal tract. We also emphasize its relevance to the immune system and highlight its powerful influence on intestinal diseases, including inflammations and tumors. In addition, we provide fresh insights into future clinical diagnostic and therapeutic strategies related to TCs.

## 1 Introduction

TCs within the rat trachea and the gastric wall were originally discovered in the 1950s (Rhodin and Dalhamn, 1956; Jarvi and Keyrilainen, 1956). Because TCs have apical brush borders, researchers may identify them based on their distinct morphological traits. As intestinal TCs were detected above the Paneth cells at the crypt (the +4 position), they were assumed to be reserved stem cells (May et al., 2008; Dekaney et al., 2009; May et al., 2009; DelGiorno et al., 2020). Due to similarities between TCs and enteroendocrine cells, it was commonly considered that TCs were a subset of enteroendocrine cells. Recent investigations, however, have revealed that TCs are post-mitotic and short-lived, implying TCs represent a distinct secretory intestinal epithelial cell (IEC) lineage different from enteroendocrine, Paneth, goblet cells (GCs), and enterocytes (Gerbe et al., 2009). Further information on post-mitotic cells is depicted in Box 1 (Gerbe et al., 2012). Their distinct requirements for biomarkers and the transcription factor testified to their uniqueness. Although rarely discussed, there are multiple TC subtypes with distinct traits and roles. The functions of this unusual cell type, however, have yet to be properly examined. As a secretory epithelial cell lineage, TCs can secrete multiple molecules that are required for the type 2 immune response against helminth and bacterial infection.

Inflammations or neoplasms may result from TC malfunction in the digestive system. The identification of one of the TCs markers in CSC raises the hypothesis that aberrant TC proliferation is linked to intestinal neoplasms, and more study is needed to validate the underlying mechanism to treat these diseases. As a result, the goal of this review is to provide a comprehensive assessment of the characteristics and functions of TCs in the digestive tract, as well as a novel strategy for future clinical practice of intestinal inflammations and tumors.

## 2 History of TCs discovery

Independent investigations have documented the existence of TCs in various hollow organs since the first identification in the mouse gastrointestinal tract (Jarvi and Keyrilainen, 1956) and rat trachea (Rhodin and Dalhamn, 1956) in the 1950s. Since then, TCs have been found in several organs of various species (Jarvi et al., 1967; Luciano et al., 1968; Meyrick and Reid, 1968; Luciano et al., 1969; Chang et al., 1986; Hofer and Drenckhahn, 1992), including taste buds, pancreas, submandibular glands, and efferent ductules of testis (Jeffery, 1983; Sato and Miyoshi, 1988; Hofer and Drenckhahn, 1992; Hofer et al., 1996). (as Table 1 shown the TCs discovery milestones).

**TABLE 1:** Milestones of TC identified in different cells and tissues.

Year	Organ/tissue	Material	Model	Technique	References
1956	Trachea/glandular stomach	Rat/Mice	Exposure to SO_2_/methylcholantren-methocel suspension, which invades the epithelium, causing intestinilisation	Electron microscopy	Jarvi and Keyrilainen (1956); Rhodin and Dalhamn (1956)
1956	Gastric wall	Mice	--	Electron microscopy	Jarvi and Keyrilainen, (1956)
1967	Lung	Rat	--	--	Luciano et al. (1969)
1968	Rectum/Fundic glands of the stomach	Rat/Canine	--/Surgical biopsies	Light microscopy/electron microscopy	Luciano et al. (1968), Hammond and Ladeur (1968)
1973	Gastrointestinal mucosa	Rat	--	SEM/TEM	Isomaki (1973)
1975	Trachea and principal bronchi	Rat	SD strain	SEM/TEM	Alexander et al. (1975)
1977	Larynx	Rat	--	RSEM	Breipohl et al. (1977)
1978	Gastric Groove and Cardia	Male albino Wistar rats	--	Microscopy, TEM, Autoradiography	Wattel and Geuze, (1978)
1979	Colon	Male adult Swiss mice	Continuous infusion of 3H-thymidine	Silver-iron hematoxylin technique, TEM	Tsubouchi and Leblond, (1979)
1979	Pancreas	Ruminants		Light/electron microscopy	Weyrauch (1979)
1981	Bile duct	Rat	--	TEM/SEM	Luciano et al. (1981)
1984	Nasal cavity	CDF^®^(F-344)/CrlBr rats	--	SEM	Popp and Martin, (1984)
1992	Testicular ductuli efferentes	Rat	--	Immunostaining	Hofer and Drenckhahn, (1992)
1996	Taste buds	Rat	--	Immunostaining, Immunoblotting, PCR, Sequencing.	Hofer et al. (1996)
1997	Submandibular gland	Male Wistar rats	--	TEM, HRP treatment, glycoconjugate cytochemistry	Sato and Miyoshi, (1988)
1998	Pancreatic	Adult Wistar rats	--	Antibodies (α-gustducin) and immunostaining, Immunoblotting	Hofer and Drenckhahn, (1998)

TCs have been discovered in human airways in pathologic conditions but not among healthy people (Gordon and Kattan, 1984; Cerezo and Price, 1985; DiMaio et al., 1988).

## 3 Morphology of TCs

Early studies in rodent models revealed that TCs possess brush boundaries formed by distinct apical bristles (Silva, 1966; Luciano and Reale, 1979; Luciano and Reale, 1997). Actin filaments sustain the microvilli, which could be visualized by phalloidin (Hofer and Drenckhahn, 1998). Since the identification of this unique cell type, researchers have given it names such as “fibrillovesicular” “peculiar” “caveolated” “brush” and “tuft”. In 2005, the term “tuft” was proposed as a moniker for this cell lineage (Reid et al., 2005). The overall morphology of TCs varies among hollow organs (Luciano and Reale, 1979; Sato, 2007), and the intestinal TC body is fashioned like a cylinder with thinner basal and apical ends (Meyrick and Reid, 1968). Although TCs in various organs have different functions, most researchers believe they belong to the same cell type.

Furthermore, using ATUM, SBF, and SEM, Hoover et al. (2017) discovered a novel tubulovesicular system in TCs. Volume rendering revealed a sophisticated network of tubules connecting the microvilli to the rough endoplasmic reticulum in TCs from the gastrointestinal tract. The tubular network may facilitate molecular interaction between TCs and the intestinal lumen or adjacent cell nuclei (Herring et al., 2018). However, unlike TCs in the alimentary tract, those in the respiratory tract lack a tubular network.

**FIGURE 1:**
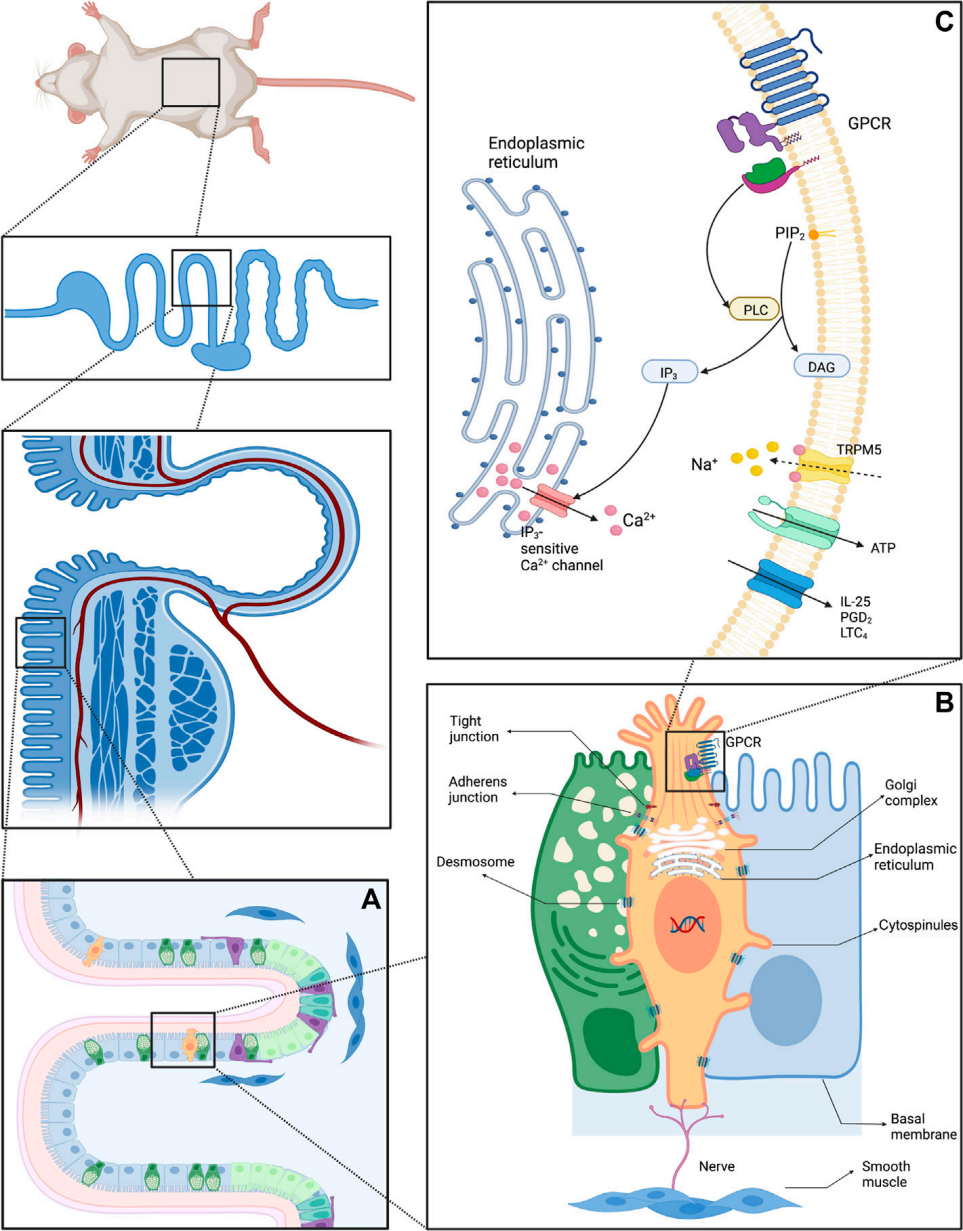
Diagram of the structure and composition patterns of IECs **(A)** Schematic diagram of Lgr5^+^ stem cells and differentiated progeny of the crypt-villus **(B)** Structure of intestinal TCs and adjacent enterocytes: Intestinal TCs possess distinguish morphological characteristics, especially the unique brush border. The cytospinules can directly contact the neighboring cells, serving as a bridge between the extracellular and intracellular environment. The tubular network within TCs can transport cargo *via* vesicles. **(C)** Schematic diagram of the vicinity of the TC cell membrane.

Aside from the well-known brush border, Luciano *et al.* discorvered lateral projections formed by basolateral membrane and microvilli in TCs. Although the protrusions might extend to neighboring cells and connect to their nuclei, transmission electron microscopy failed to corroborate the details (Luciano and Reale, 1979; Luciano and Reale, 1990). Hoover et al. (2017) revealed the nanostructure of TCs using ATUM, SBF, and SEM and dubbed the protrusions “cytospinules.” Every TC has three or four cytospinules in direct contact with the nuclear membranes of neighboring cells. Since the specific function of cytospinules has been vague, it is speculated that this direct cell-to-cell interaction might play a substantial role in intercellular comminucation (Figure 1 depicts structure and composition patterns of intestinal TCs). Moreover, although secretory cells are scarcely distributed in the intestinal epithelium, TCs may exist close to other secretory cells. It is now hypothesized that GCs receive cytokine signals when secreting mucus and expelling helminths during the “weep and sweep” process. If a TC is adjacent to a GC, would it generate a direct signal to rapidly promote GC’s role in “weep and sweep”? The detailed signaling pathway has yet to be demonstrated.

## 4 Intestinal TCs originate from Lgr5^+^ stem cells

TCs account for around 0.4% of the IECs in the murine alimentary tract (McKinley et al., 2017). TCs, in contrast to GCs, decrease progressively from the jejunum to the colon, peaking in the proximal small intestine, according to earlier research. Because the small intestine performs an important absorptive role and contains the majority of the TCs, it may be assumed that TCs are associated with intestinal absorption (Cheng et al., 2018). The differentiated TCs first appear around the 7 postnatal day and can be promptly detected a week later, relying on self-renewal stem cells.

Tsubouchi and Leblond’s experiment in the 1970s offered a vital insight into the TCs progenitors. The label first developed in the enteroendocrine cells at the base of the crypts after ^3^H-thymidine infusion began, and then traveled to the “+4 position,” where the TCs resided (Tsubouchi and Leblond, 1979). Genetic tracing experiments using Cre-activable Rosa26-LacZ reporter mouse and the Lgr5^EGFP−IRES-CreERT2^ mouse have demonstrated that Lgr5^+^ crypt basal columnar (CBC) cells can self-renew and differentiate into diverse cell lineages over time, implying that Lgr5^+^ CBC cells possess characteristics of stem cells and that TCs are generated from Lgr5^+^ stem cells (Gerbe et al., 2011). Yui et al. (2012) further testified the cellular origin of TCs by cultivating organoids derived from single Lgr5-EGFP cells.

It is hypothesized that intestinal stem cells (ISCs) dwell in the crypt and are continually feed differentiated progeny from the crypt to the villi. Although most differentiated daughter cells are phased out after a short existence of 3–5 days, long-lived ISCs can self-renew. Initially, cells with preserved labels at the +4 position of the crypt were thought to represent ISCs (Rea et al., 1975). Recent lineage tracing investigations, however, have discovered that CBCs positioned at locations +1 to +3 in the crypt are quickly cycling, self-renewing ISCs (Barker et al., 2007).

Although we have long assumed that the differentiation of stem cells is irreversible based on our comprehension of the hematopoietic system, a series of studies have shown that mature intestinal epithelial cells (IECs) show a strong capacity for retro‐differentiation, indicating that IECs have more remarkable plasticity (de Sousa and de Sauvage, 2019). A more dynamic model, rather than the traditional view of the stratified organization of the gut, is thus better suited to explain this phenomenon, in which various differentiated cells within the intestinal epithelium can dedifferentiate and function as an alternative source of stem cells in inflammation and tumorigenesis. IECs differentiation is regulated by the interaction of growth and developmental stimuli, metabolites, and signaling pathways such as Notch, EGF, BMP, and Hippo signaling.

## 5 TCs qualify as a distinct secretory epithelial cell

DCLK1^+^ cells have long been considered to be quiescent stem cells (Giannakis et al., 2006; May et al., 2008; Dekaney et al., 2009; Jin et al., 2009; Sureban et al., 2009) and a subset of enteroendocrine cells (Formeister et al., 2009; Kokrashvili et al., 2009). However, with further studies of TCs’ characteristics, it has been confirmed that DCLK1^+^ TCs are a particular type of IECs (Gerbe et al., 2011).

### 5.1 Classification of differentiated IECs

**FIGURE 2:**
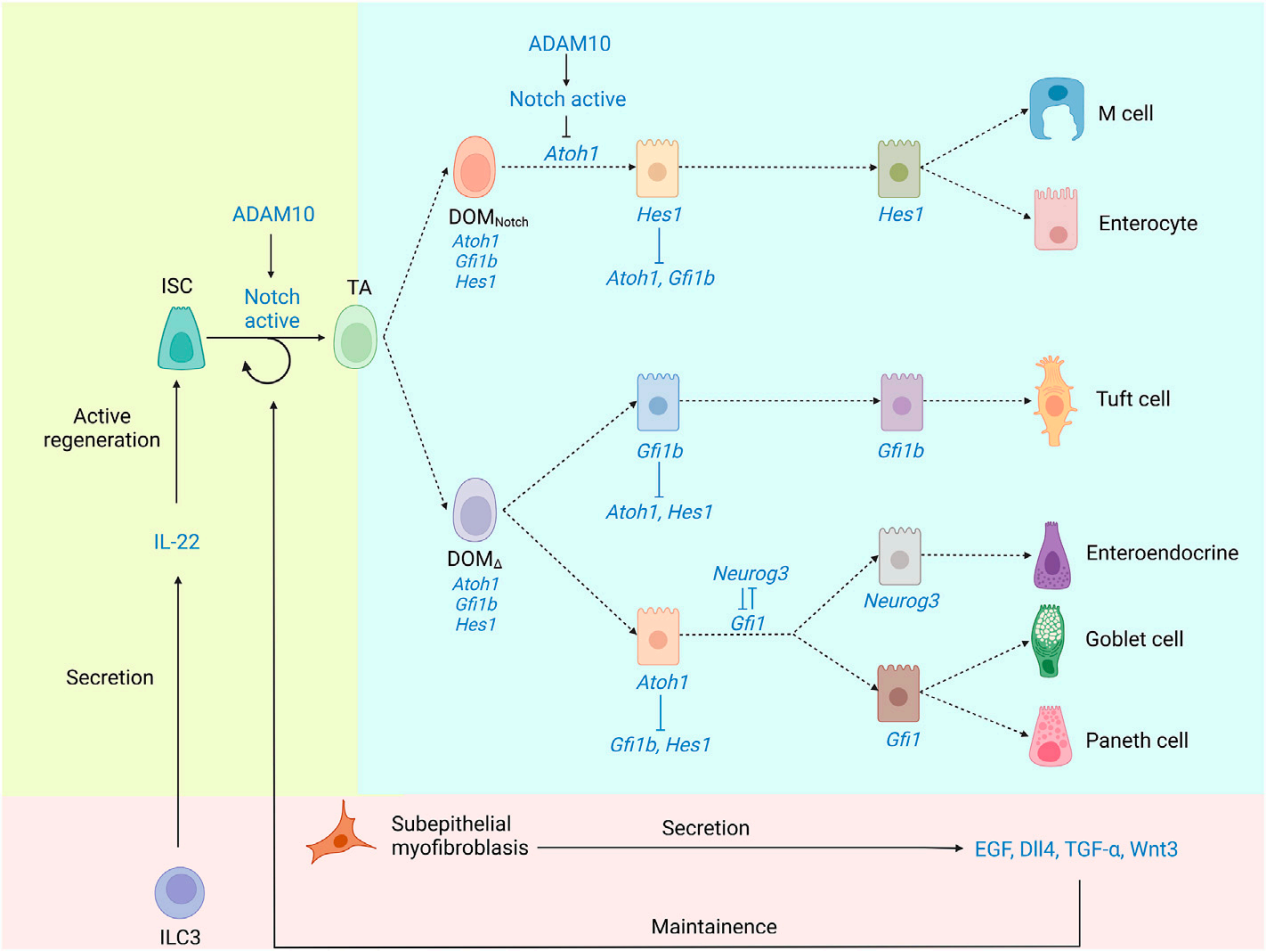
The underlying mechanism of IEC differentiation. Lgr5^+^ CBCs generate TA progenitor cells, which then differentiate into a spectrum of different absorptive and secretory cell lineages. Notch signaling is crucial to the maintenance of ISCs and the differentiation of TA. ADAM10, as an α-secretase, promotes Notch signaling. *Atoh1* is necessary for stem cells to differentiate into secretory cells, while *Hes1* acts to repress secretory cells. There is reciprocal repression between *Hes1* and *Atoh1*. TCs are produced from Gfi1b-expressing progenitors. Progenitors differentiate into three distinct cell fates through the guidance of three characteristic transcription factors, *Hes1*, *Atoh1*, and *Gfi1b*. Although TC is not *Atoh1*-dependent, TCs are hypothesized to derive from secretory progenitors.

The small intestine contains crypt-villus units that repeat. The pioneering experiments of Barker et al. (2007) demonstrated that Lgr5^*+*^ CBC stem cells are the progenitors of a variety of epithelial cells, which inhabit the base of the crypt and are intercalated between Paneth cells. Lgr5^+^ CBCs rapidly create transit-amplifying (TA) progenitor cells that move upwards and completely develop before entering the crypt (Barker et al., 2007). Based on morphology and expression features, differentiated epithelial cells may be generally separated into two types: secretory cells and absorptive cells (Flier and Clevers, 2009). Although as many as seven lineages of cells have been described in the intestinal epithelium, including cup cells and “membranous” (M) cells (Madara, 1982; Neutra, 1998), only five of which are usually considered. (Figure 2 depicts an IEC differentiation diagram).

Absorptive epithelial cells comprise the majority of differentiated epithelial cells, while secretory cells account for only 1%. Absorptive IECs play various roles in digestion, nutrition absorption, and mucosal defense. Secretory IECs are in charge of secreting antimicrobial peptides and growth factors, as well as the controlling the gut flora and surrounding stem cells (Gerbe et al., 2011).

### 5.2 Using transcription factors to identify TC from other IECs

Previous research has emphasized the transcriptional start sequence, the participation of particular transcription factors, and epigenetic modification. It is assumed that a multitude of mechanisms is involved in IEC differentiation, however, it is disputed whether transcriptional modulation is involved.

#### 5.2.1 Lateral inhibitory notch signaling in IEC fate decisions

The Notch pathway is one of the critical signaling pathways in maintaining the balance of epithelial cell proliferation and differentiation (Kimble and Simpson, 1997; Shen et al., 2004), which is best known for specifying different cell fates of neighboring cells *via* an evolutionarily conserved process of “lateral inhibition” (Chitnis, 1995).

##### 5.2.1.1 The contribution of notch signaling in intestinal homeostasis and cell fate decision

Notch signaling is crucial to the maintenance of ISCs and the differentiation of TA progenitors. To maintain the stem cell pool, the Notch signaling pathway operates directly on intestinal stem cells. It also regulates the differentiation of the secretory and absorptive cells through “lateral inhibition” (Sancho et al., 2015).

Notch “active” TA progenitors are destined to be absorptive progenitors, in which Notch targets the *Hes/Hey* transcription factors, repressing the expression of *Atoh1* and *Dll-1/4* ligand (Akazawa et al., 1995; Jensen et al., 2000; Yang et al., 2001). These cells would ultimately differentiate into post-mitotic enterocytes after several rounds of proliferation. Notch “low” TA progenitors are destined to be secretory progenitors, in which low Notch activity disinhibits the expression of *Atoh1* and *Dll-1/4* ligand. These cells would rapidly differentiate into distinct secretory cell types (Jensen et al., 2000; Yang et al., 2001; Bjerknes and Cheng, 2005). In summary, using lateral inhibition, Notch promotes differentiation towards the absorptive lineage, whereas the Notch-low state permits differentiation towards the secretory cell lineage.

##### 5.2.1.2 ADAM10 regulates notch signaling

As a family member of Sultidomain, a Disintegrin and Metalloproteinases (ADAMs) are involved in signal transduction processes that regulate cell migration and adhesion proteolysis (Hartmann et al., 2002). Analysis has demonstrated that ADAM10 is an α-secretase that promotes Notch signaling. The systemic *Adam10*-deficient mice embryos die at E 9.5 due to defective in somatic cell development, angiogenesis, and neurogenesis, similar to those of the Notch-defective mice (Hartmann et al., 2002).

##### 5.2.1.3 Notch-signaling driven ternary switching and Gfi1b-expressing progenitors

Secretory cells are more common in *Hes1*-deficient epithelial cells than absorptive cells, according to Bjerknes et al. (Jensen et al., 2000), suggesting that *Hes1* represses secretory cells, potentially *via* suppressing the expression of *Atoh1* (Akazawa et al., 1995; Jensen et al., 2000; Yang et al., 2001). Therefore, the reciprocal inhibition between *Hes1* and *Atoh1* would lead DOM (daughters of TA progenitors/daughters of Mix) to pass through a binary switch *via* the Notch signaling (Fortini, 2009). (Box 2 depicts alternate nomenclature for cells in the differentiation process as well as their relationship).

Unfortunately, this model does not include TCs. Contrary to Gerbe *et al.*, Bjerknes *et al.* discovered that conditional *Atoh1* deletion dramatically increases TC populations, suggesting that the differentiation and survival of TCs are independent of *Atoh1,* but Atoh1 may be transiently expressed in TA cells before lineage commitment (Bjerknes et al., 2012). TCs are derived from progenitors that express Gfi1b. These findings support a model in which progenitors develop into three discrete cell fates under the control of three separate transcription factors, *Atoh1, Hes1*, and *Gfi1b*. Notch signaling leads *Hes1* to dominate one of the two major differentiated cell lineages, producing absorptive cells. Correspondingly, *Atoh1* or *Gfi1b* dominate the other major cell lineage, producing secretory cells or TCs, resulting in a ternary switch for cell fate determination (Bjerknes et al., 2012). The schematic diagram of the ternary switch is shown in Figure 2. TCs require another transcription factor to differentiate than enteroendocrine, GCs, and Paneth cells; non-etheless, Bjerknes *et al.* argue that these four lineages share many properties and also hypothesized that the secretory progenitor gives birth to the TC lineage.

To conclude, *Atoh1*, *Hes1*, and *Gfi1b* are components of a genetic network that forms a ternary switch in the TCs *via* Notch signaling.

##### 5.2.1.4 The debatable regulation of TCs by ATOH1

Although the significance of ATOH1 in regulating enteroendocrine, Paneth, and goblet cells is well established (Yang et al., 2001), how ATOH1 regulates TCs remains controversial (Gerbe et al., 2011; Gracz et al., 2018). Recent cell lineage tracing investigations have revealed that ATOH1^+^ cells contain stem cell characteristics and facilitate epithelial regeneration following damage (Ishibashi et al., 2018; Tomic et al., 2018). Moreover, the *Atoh1* ^−/−^ mouse model published by Banerjee *et al.* showed that although colonic TCs depend on ATOH1, TC expansion can be observed in small intestinal in *Atoh*KO mice, contradicting the conclusion that TCs are dependent on ATOH1 found in a prior work by Gerbe et al. (2011). It was inferred that a subset of small intestinal TCs may be independent of ATOH1 and follow a distinct path of development (Herring et al., 2018). Banerjee *et al.* demonstrate that ATOH1-independent TCs expand through a metabolic communication network during luminal microbiota perturbations, a specific mechanism that could be used to suppress inflammation and repair the epithelial damage caused by Crohn’s disease (CD) (Banerjee et al., 2020).

#### 5.2.2 Other transcription factors involved in the TC differentiation

Notch regulates the cell fate decisions of TA progenitors by influencing the essential transcription factor *Atoh1* (Fre et al., 2009; VanDussen et al., 2012). These events are coordinated by Notch1/2 receptors and DLL1/4 (Riccio et al., 2008; Pellegrinet et al., 2011; Carulli et al., 2015). *Atoh1* target genes, such as the SAM pointed domain containing Ets transcription factor (*Spdef*) genes, Kruppel-like factor 4 (*Klf4*), SRY-box containing gene 9 (*Sox9*), *Neurog3*, and growth factor-independent 1 (*Gfi1*), are responsible for secretory cell type specification. Although evidence shows that Paneth cells and GCs have a common ancestor, it is unclear how multipotent secretory progenitor cells form particular secretory cell types (Barker, 2014; Sancho et al., 2015). Wnt signaling facilitates Paneth cell development by directly activating lineage-specific transcription factors and differentiation genes such as *defensins* (van Es and Clevers, 2005; van Es et al., 2005; Farin et al., 2012; Kim et al., 2012; San Roman et al., 2014). However, since this Wnt signaling pathway was suppressed, the Lgr5^+^ CBC stem cells could not be observed (Korinek et al., 1998; Pinto et al., 2003).

Therefore, Notch and Wnt activity must always be in balance with each other to ensure the survival of intestinal stem cells, the proper development of all types of epithelial cells, and the maintenance of crypt stability and intestinal function. *Sox9*, a Wnt signaling target, is expressed in crypt epithelial cells. TCs occur in *Sox9*-deficient intestinal epithelium due to adequate Sox9 expression inside differentiated TC (Bastide et al., 2007; Mori-Akiyama et al., 2007). Growth factor-independent 1b (*Gfi1b*) is expressed at greater levels among Trpm5-expressing TCs, according to a transcriptome comparison (Bezencon et al., 2008). The growth factor-independent 1 (*Gfi1*) is detected in goblet and Paneth cells (Bjerknes and Cheng, 2010), where it may block the transcription factor of the enteroendocrine cells (*Neurog3*) (Jenny et al., 2002; Mellitzer et al., 2010). TCs are also dependent for their development on the transcription factor *Pou2f3*. *Pou2f3* ^−/−^ mice lack intestinal TCs and have defective mucosal type 2 responses to helminth infection (Gerbe et al., 2016). The detailed requirements are listed in Table 2.

**TABLE 2:** Summary of the transcription factors involved in TCs and other secretory IECs.

Transcription factors	Cell types				References
	Tuft	Paneth	Goblet	Enteroendocrine	
*Atoh1*	?	Required	Required	Required	Yang et al. (2001), Shroyer et al. (2007), van Es et al. (2010)
*Neurog3*	--	--	--	Required	Gerbe et al. (2011), Jenny et al. (2002), Mellitzer et al. (2010)
*Gfi1*	Expressed	Required	Required	--	Bjerknes and Cheng, (2010), Bezencon et al. (2008)
*Sox9*	Expressed	Required	--	--	Bastide et al. (2007), Mori-Akiyama et al. (2007)
*Klf4*	--	--	Required	--	Katz et al. (2002)
*Spdef*	--	Required	Required	--	Gregorieff et al. (2009), Noah et al. (2010)

### 5.3 Biomarkers of TCs

The lack of identifiable biomarkers has hampered the study of TCs since their discovery in the 1950s. Identifying more viable unambiguous, and specific markers has enhanced the research situation, allowing for a more detailed examination of TCs. With updated biomarker information, far more complete research is expected.

#### 5.3.1 Ambiguous markers of TCs

Cytokeratin 18 (Hofer and Drenckhahn, 1996), Ulex europaeus lectin 1 (Gebhard and Gebert, 1999; Gebert et al., 2000), neuronal nitric oxide synthase (Kugler et al., 1994), Villin, and fimbrin (Hofer and Drenckhahn, 1992), are either expressed ubiquitously in the intestinal epithelium, or also expressed in TCs within limited areas. (Kugler et al., 1994; Gebert et al., 2000; Jang et al., 2007; Sutherland et al., 2007; Bezencon et al., 2008; Kokrashvili et al., 2009). Taste-related biomarkers such as α-gustducin, β-endorphin, uroguanylin, and Met-Enkephalin (Perez et al., 2002; Bezencon et al., 2007; Kaske et al., 2007) are expressed within TCs. They may engage in the taste transduction (Hofer et al., 1996; Hofer and Drenckhahn, 1998). However, the exact relationship between these biomarkers and TC is not elucidated, so these markers are considered to be TCs non-specific. Given that TCs secrete such a wide variety of proteins, the complexity of TC’s functions may be far beyond our imagination. Therefore, the study of TC function is very promising and may provide us with enlightenment in many aspects.

#### 5.3.2 Specific markers of TCs

Although TRPM5 (transient receptor potential cation channel, subfamily M, member 5) is expressed by enteroendocrine cells (Bezencon et al., 2007), it is one of the best markers for TCs, since Trmp5-expressing IECs are primarily TCs (Kaske et al., 2007) and the detailed depiction of TRPM5 is shown in Box 3.

All TCs, characterised by DCLK1 and Growth factor independent 1b (GFI1b) expression also co-expressed the Pou domain, class 2, transcription factor 3 (POU2F3) (Bjerknes et al., 2012; Gerbe and Jay, 2016). TCs also express the cyclooxygenase 1 (COX1) and cyclooxygenase 2 (COX2) enzymes (Bezencon et al., 2008). May et al. (2014) discovered that DCLK1^−/−^ mice had altered gene expression profiles of growth and functions in TCs, proving the relevance of DCLK1 in TCs. DCLK1^+^ cells were formerly assumed to represent latent intestinal epithelial stem cells (Giannakis et al., 2006; May et al., 2008; Dekaney et al., 2009; Jin et al., 2009; May et al., 2009; Sureban et al., 2009). However, it was later proven to be untrue. Gerbe *et al*. discovered that DCLK1^+^ cells were distributed throughout the adult mouse’s intestinal epithelium, but only 21% of the TCs were identified in the crypt (Gerbe et al., 2009). This evidence revealed that the DCLK1^+^cells do not belong to stem cells. DCLK1 did not co-stain with any known markers of Paneth cells, enterocytes, GCs, or enteroendocrine cells, above which gives direct proof that the DCLK1 represented a gene signature of the intestinal TCs in mice (Bezencon et al., 2008). A 2019 study showed that DCLK1 is connected to the activation status of TCs. Still, it was not engaged in TCs growth (Yi et al., 2019), which was related to the response to intestinal epithelial damage (May et al., 2014; Westphalen et al., 2014; Qu et al., 2015). Gerbe *et al.* validated DCLK1 as a particular marker of post-mitotic TCs in the mouse intestinal epithelium based on repeated co-staining experiments and micro-array data (Bezencon et al., 2008; Gerbe et al., 2009). The data indicate that DCKL1^+^ intestinal cells are TCs rather than long-lived quiescent stem cells. Special attention should be paid to SUCNR1 expressed exclusively in mice, confirming *Sucnr1* as a TC gene signature (Lei et al., 2018).

Cells expressing DCLK1, hematopoietic prostaglandin-D synthase (HPGDS), COX1, COX2, and SOX9 have more significant immunoreactivity to F-actin, -tubulin, and villin. These properties resemble the typical TC trait (May et al., 2014). The evidence presented above shows that DCLK1, HPGDS, COX1, COX2, and SOX9 coexpression is confined to TCs in the epithelium.

To summarize, whereas enterocytes, Paneth cells, enteroendocrine cells, and GCs are derived from Lgr5^+^ CDC stem cells, TCs constitute a unique cell lineage with distinct transcription factor requirements and biomarkers. Apart from *Atoh1*, TC differentiation is unaffected by other transcription factors in other epithelial cells. There are no shared markers between TCs and other epithelial cells.

## 6 Subtypes of TCs

Immunostaining of combination markers and morphological inspection are now widely employed to identify TCs; nevertheless, minor changes between TC subtypes cannot be detected with this approach. We do not yet know all of the indicators that can distinguish cell subtypes in the gut. We analyzed multiple standards for classifying TC subsets using various criteria that may aid future studies.

### 6.1 DCLK1/5HT-IR cells represent a novel subtype of TCs

According to double immunostaining evidence, DCLK1/5HT-IR cells contain serotonin (5HT) and are a novel subtype of DCLK1-immunoreactive (IR) TCs. These cells shrank distally from the small to the large intestine. 5-HT has a wide range of biological roles, including cognition, learning, memory, emotional control and vasoconstriction (Young, 2007). Approximately 90% of the serotonin in the human body is located in the enterochromaffin cells of the GI tract, where it also involves in the accommodation of gut homeostasis (Berger et al., 2009). In a word, DCLK1/5HT-IR cells, as a non-negligible neo-subtype of TCs, may contribute to the intestinal physiological function (Cheng et al., 2019).

### 6.2 Tuft 1 and Tuft 2

Transcriptome analysis revealed two additional TC subgroups: neuronal TCs (tuft 1) and immunological TCs (tuft 2). Despite the fact that DCLK1 and IL-25 are expressed by both TC subtypes, their roles are distinct (Haber et al., 2017). Tuft 1 has higher levels of neuronal gene expression profile, including *Ninj1*, *Nrep*, and *Nradd*. Immunological genes, such as those encoding CD45 and thymic stromal lymphopoietin (TSLP), were expressed at higher levels in tuft 2 (Haber et al., 2017). When parasite infections occur, tuft 2 outnumbers tuft 1 to form the majority of mouse gastrointestinal TCs (Haber et al., 2017).

According to a 2020 research, there may be another subtype of TC that mimics intestinal endocrine cells following the treatment of scopolamine (Middelhoff et al., 2020). The properties and functions of this novel subtype TCs need to be investigated urgently.

### 6.3 ATOH1-dependent and ATOH1-independent TCs

Banerjee et al. (2020) identified heterogeneous TC populations that respectively undergo ATOH1-dependent and ATOH1-independent pathways. Both ATOH1-independent and dependent TCs can be observed in the small intestine, but only ATOH1-dependent TCs can be observed in the colon. Banerjee *et al*. also found that ATOH1-independent TCs are a flexible cell population that can expand in the presence of luminal perturbations, whereas the ATOH1-dependent cell population is constant. Specifically, succinate drives ATOH1-dependent TC gene expression and growth in symbiotic bacteria (Stumhofer et al., 2006; Langille et al., 2013).

## 7 Functions of TCs

Chemosensory cells are used by most organs to respond to changes and maintain homeostasis. TCs are responsible for chemoreception and secretion, which includes sensing and processing chemical signals as well as mending the epithelium (Chandrakesan et al., 2016).

### 7.1 Biologically active molecules released from TCs

Previous research has revealed that TCs release various chemicals, including NO, leukotrienes, IL-25, opioids, fatty acid metabolism-related proteins, and components of the eicosanoid pathway. These molecular secretions demonstrate that TCs may perform a variety of roles in the digestive tract, as summarized in Table 3. These secretion-related activities might provide deeper insight into inflammation and tumor-related pathways. Box 4 depicts the function of TCs in various organs or tissues.

**TABLE 3:** Molecule secretion of gastrointestinal TCs.

Molecules expressed by TCs	Potential functions of TCs	References
Nitric oxide (NO), leukotrienes, prostaglandins, IL-25	GC and TC hyperplasia in inflammation and injury; ILC2 activation and IL-13 secretion	Hass et al. (2007), Sbarbati et al. (2010), Schutz et al. (2015), Gerbe et al. (2016), Nadjsombati et al. (2018)
Opioids	Intestinal secretion, gut motility, gastric emptying; pain, paresthesia, and emotion	Holzer (2009), Kokrashvili et al. (2009)
Fatty acid metabolism-related protein	Fatty acid sensing or absorption	Iseki et al. (1991), Iseki and Kondo, (1990)
Members of the eicosanoid pathway	Smooth muscle contraction	Bezencon et al. (2008)

### 7.2 Chemoreception

Changes in nutrition, pH, and microbiota can be detected by TCs, which are found in the airway and digestive tract. Because of their physical similarities to lingual taste bud cells, TCs were assumed to have a role in chemoreception. Members of the pancreatic and intestinal taste transduction pathways support this theory (Hofer et al., 1996; Hofer and Drenckhahn, 1998). TCs express several signaling molecules, including α-gustducin (also known as the guanine nucleotide binding protein alpha transducing 3, or GNAT3) (Hofer and Drenckhahn, 1998), TRPM5 (Kaske et al., 2007), G protein-coupled taste receptor type 1 member 3 (TAS1R3), the calcium signal transducer phospholipase Cβ2 (PLCβ2) (Ogura et al., 2010), β-endorphin, uroguanylin, and Met-Enkephalin, (Perez et al., 2002; Bezencon et al., 2007; Kaske et al., 2007). According to some study, TCs are a component of the diffuse chemosensory system (Sbarbati and Osculati, 2005). Furthermore, succinate receptor 1 (SUCNR1) was found to be expressed in TCs, and Lei et al. (2018) identified SUCNR1 as a TC-specific marker in mice, suggesting that SUCNR1 might aid in detecting infectious pathogens, triggering the proliferation of TCs and GCs involving in type 2 immune response.

### 7.3 Tuft-ILC2 circuit mediated helminth expulsion pathway

Helminth infection is still regarded as a major worldwide health issue by scientists and practitioners, owing to its widespread occurrence and severe societal effect, particularly in less developed countries and regions. However, the early sensing and signaling mechanisms that initiate type 2 immunity against helminths remain unclear. The identification of these pathways might pave the way for the development of vaccines and medicines that target type 2 immunity. A recent study found that helminth infection can cause the synthesis of immunoregulatory substances that attract immune cells, resulting in infestations and inflammatory responses (Lightowlers and Rickard, 1988). Nontheless, the fundamental process, as well as the chemicals and cells involved, remain unclear. TCs were previously unseen to have great importance in this immunoreaction. TCs have been discovered as a significant activator of type 2 immunity in the small intestine by three distinct groups during the last decade (Gerbe et al., 2016; Howitt et al., 2016; von Moltke et al., 2016). Through a chemosensory mechanism, TCs in the small intestine detect helminths such as *Heligmosomoides polygyrus*, *Trichinella spiralis*, *Nippostrongylus brasiliensis*, and various species of *Tritrichomonad protists.*

In response to helminth infection (such as *H. polygyrus*), impaired epithelial cells release mediators such as leukotrienes, IL-22, and IL-33 (Artis and Grencis, 2008). Upon detecting the ligand, TCs transmit signals to the underlying lamina propria’s group 2 innate lymphoid cells (ILC2s), evoking an inflammation response. TCs are the only cell lineage that expresses IL-25 continuously (von Moltke et al., 2016). IL-25 stimulates ILC2s *via* the IL-17RB receptor. However, studies have observed that parasite-secreted *H. polygyrus* alarmin released inhibitor (HpARI) could hamper the “weep and sweep” immune response by limiting the IL-33 synthesis from injured epithelial cells (Osbourn et al., 2017). When subjected to helminth chemosensing, TCs produce cysteinyl leukotrienes (cysLTs), which rapidly activate type 2 immunity, accordng to McGinty *et al.*. CysLTs in collaboration with IL-25 stimulate ILC2s, and TC-specific leukotriene synthesis suppresses type 2 immunity and delays helminth clearance (McGinty et al., 2020). ILC2 activation may acquire additional signals to regulate the circuit in addition to IL-25. TCs in the colon, unlike those in the small intestine, respond to bacteria rather than parasites. Bacterial microflora can control colonic TC populations and stimulate TC growth, whereas colonic TCs have been shown to inhibit bacterial penetration and promote epithelial repair (McKinley et al., 2017; Wilen et al., 2018; Yi et al., 2019; Banerjee et al., 2020).

As a member of the chemokine family, IL-13 could stimulate secretory epithelial cells proliferation to boost mucus production and promote smooth muscle contraction to expel parasites in the intestine (Kamal et al., 2002; Gerbe et al., 2016; Howitt et al., 2016; Sharpe et al., 2018). IL-13 signals act directly on ISCs and bias their development towards the TCs and GCs, resulting in proliferation and a feed-forward loop in the tuft-ILC2 circuit. Upon the process, the quantity of TCs might rise tenfold within a few days of parasite infection (Gerbe et al., 2016; von Moltke et al., 2016). Given that IL-4 and IL-13 share a component, IL-4R, they may promote the proliferation of TCs (Gerbe et al., 2016), which also initiate smooth muscle contraction by releasing acetylcholine (ACh) and facilitate TCs to expel worms (Jonsson et al., 2007).

**FIGURE 3:**
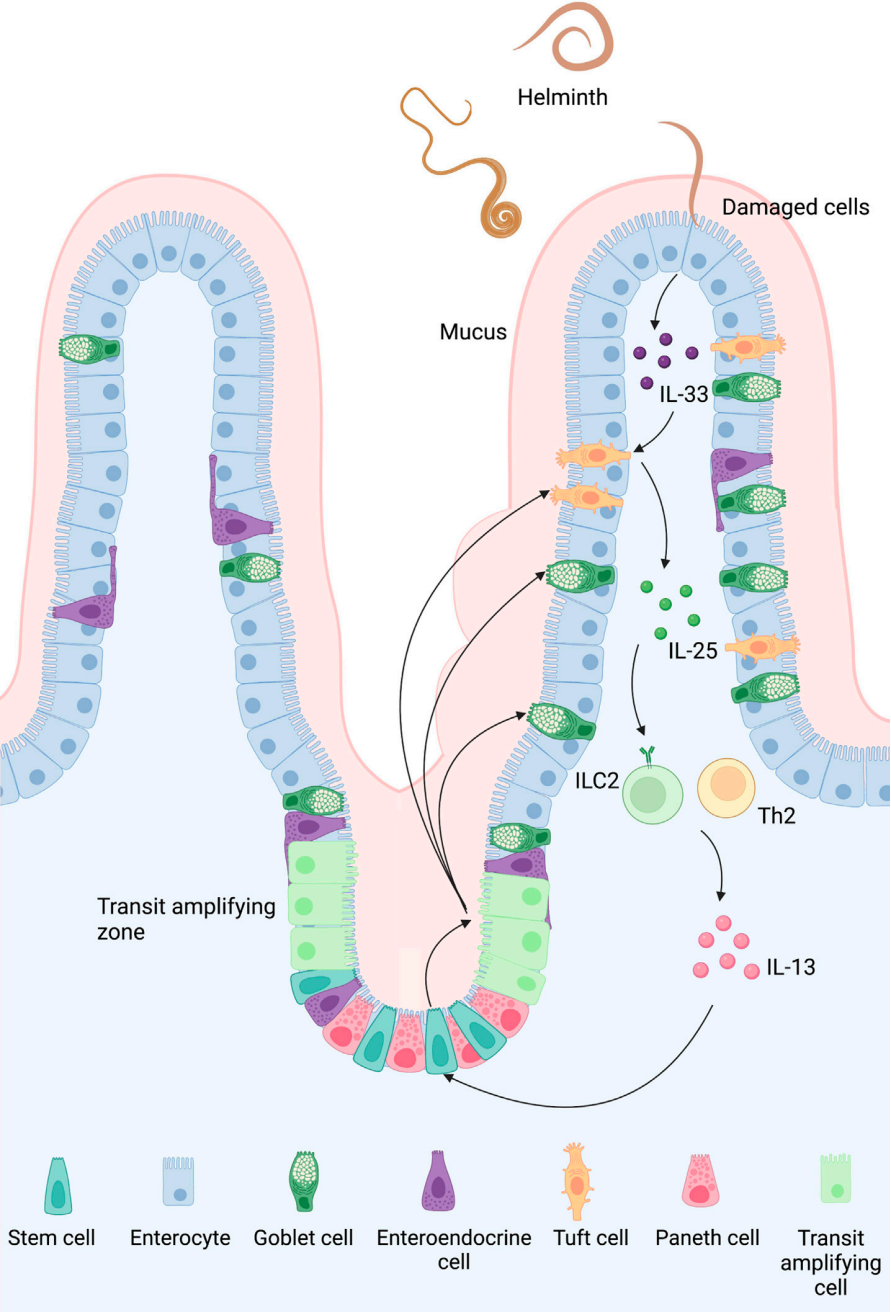
Type 2 immune response orchestrated by TCs in the small intestine: A feed-forward loop of the tuft 2-ILC2 axis can be observed in the early stage of intestinal helminth infection. Once the epithelial cells are damaged by a helminth, they release IL-33 and trigger TCs to secrete IL-25. Activated by these cytokines, ILC2 then produces IL-4 and IL-13, which promotes goblet and tuft hyperplasia and smooth muscle contraction. Although the exact mechanism of how TCs sense an infection in the first place is still unclear, TCs might sense succinate secreted by helminth and bacteria *via* protein-coupled succinate receptor SUCNR1. An intracellular Ca^2+^ flux follows the signal and opens the cation channel TRPM5, causing a Na^+^ influx which depolarizes TCs.

In mouse models, intestinal TCs appear around two weeks after birth, coinciding with epithelial changes in metabolic and nutritional behavior (Gerbe et al., 2011), as well as ILC2 and ILC3 growth (Hoyler et al., 2012), and the formation of solitary lymphoid clusters in the gut (Kiss et al., 2011). This research reveals a relationship between the ILC-epithelial cell axis and metabolic adaptation, showing that the innate immune system is important in homeostasis. A better knowledge of the innate immune system might pave the way for potential immunological advancements. The process of type 2 immune response orchestrated by TCs is shown in Figure 3.

### 7.4 GPCR-PLCγ2-Ca^2+^ signaling axis involved in the elimination of bacterial infection *via* TCs

Researchers revealed in 2022 that, in addition to their role in the immunological response to helminth infection, TCs (tuft 2) also contribute to bacterial clearance *via* a Vmn2r26-mediated mechanism. Animals lacking CD45^+^ tuft 2 were more vulnerable to pathogenic bacteria, indicating that tuft 2 might develop and respond to harmful bacteria. Tuft 2 was also shown to recognize the microbial chemical N-undecanoylglycine *via* its vomeronasal receptor Vmn2r26, which can activate the GPCR-PLC2-Ca^2+^ signaling axis and produce prostaglandin D2 (PGD2), causing GCs to generate mucus and increases gut immunity (Xiong et al., 2022).

### 7.5 Potential role of TC in the amelioration of IBD

Inflammatory bowel disease (IBD), also known as ulcerative colitis (UC) and Crohn’s disease (CD), is a chronic inflammatory illness characterized by inflammation and mucosal destruction that threatens the intestine’s integrity. The primary objective of IBD treatment is to repair the inflammatory mucosa, which improves clinical symptoms, decreases disease recurrence, and increases survival without resection (Pineton de Chambrun et al., 2010; Colombel et al., 2011; Neurath and Travis, 2012).

TCs serve as important sentinels in the intestine, directing host responses to particular injuries, including helminth infection (Gerbe et al., 2016; Grencis and Worthington, 2016; Howitt et al., 2016; von Moltke et al., 2016; Gerbe and Jay, 2016), as well as facilitating epithelial repair after tumorigenesis and acute injury (Westphalen et al., 2014).

Although helminth infection itself is a global health issue, it may have an impact on the treatment of CD (Summers et al., 2005; Broadhurst et al., 2010). It is widely accepted that anti-parasitic immune responses can neutralize CD’s pro-inflammatory signals (Summers et al., 2005). Banerjee et al. (2020) observed a reduction in the number of TCs in ileal tissues of mouse models and CD patients, therefore they postulated that TCs could act as a hub between parisites and the host, thus can be used to counteract pro-inflammatory signals in the gut. In pathological situation, the absence of TCs and DCLK1 causes a regeneration deficiency, resulting in impaired recovery of the epithelium (Yi et al., 2019). In addition, the helminth-induced tuft-ILC2 loop promotes mucus secretion by GCs and TCs and protects the intestinal mucosa, which may contribute to alleviating the symptoms of IBD. To conclude, TC is a clinically feasible strategy for reducing IBD symptoms and prognosis.

### 7.6 DCLK1 is protective against radiation enteritis and DSS enteritis

Radiotherapy has become a popular treatment in many cancers, although it has certain unavoidable adverse effects. Chronic radiation enteritis has been documented in up to one in every five patients treated with pelvic irradiation, with the real number being greater (Daly et al., 1989; Yeoh et al., 1993; Miller et al., 1999; Ooi et al., 1999). Colonic inflammation should not be ignored as it is one of the key factors for colon cancer (Kim and Chang, 2014). Experiments showed that DCLK1 ablation in the intestinal epithelium worsens the outcome during acute intestinal injury induced by radaition and dextran-sodium sulfate (DSS), since inadequate DCLK1 promotes protective intestinal epithelial regeneration (May et al., 2014; Qu et al., 2015). These findings prove that DCLK1 maintains integrity of the intestinal epithelial barrier and modulates the inflammatory response (Qu et al., 2015).

### 7.7 TCs involve in regulating satiety and energy metabolism

TCs are assumed to be engaged in the gut-brain axis and metabolic control due to their closeness to metabolic-regulating enteroendocrine and enteric neurons in the gut (Cheng et al., 2018). Although the underlying mechanisms of TC participation are unknown, intestinal TCs boost secretory ability while suppressing absorptive capacity during type 2 immune response, indicating that TCs are engaged in satiety mice and energy metabolism. Furthermore, the population of TCs rises in starved mice and persists even after refeeding (McKinley et al., 2017). Evidence above suggests that TCs may aid in adapting to various dietary situations (Arora et al., 2021).

## 8 Diseases linked to TCs

A deeper understanding of the properties and functions of TCs may bring insights into studies of TC dysfunctions. Aberrant TC numbers and secretory behaviour have been observed in inflammation, infection, and tumors of the GI tract in both mice and humans (Saqui-Salces et al., 2011). TCs are normally quiescent but can be induced to proliferate in response to inflammatory stimuli (Westphalen et al., 2014; Middelhoff et al., 2017). When TCs poliferate, they may acquire mutations from stem cells and commence cancer when exposed to inflammation and damage (Westphalen et al., 2014). When the secretory behavior of TC changes, the downstream pathways are misregulated and ultimately lead to diseases. The precise processes and cause-and-effect link between TC anomalies and illnesses, however, remain unknown.

### 8.1 TC as a potential target of MNV

MNV is the primary cause of acute viral gastroenteritis worldwide, with similar incidence in high and low-income nations (Mans, 2019). Evidence shows that TCs is the principal target of chronic MNV in both the small and large intestines and may enhance immune evasion (Baldridge et al., 2015; Tomov et al., 2017; Wilen et al., 2018). In mice, TCs express high levels of MNV receptor CD300lf, which acts as a target for viral infection. Viral shedding occurs several weeks after the acute phase of infection (Teunis et al., 2015; Wilen et al., 2018).

### 8.2 Underlying linkage of IBD and TC-secreted IL-25

IBD is a collection of chronic idiopathic inflammatory illnesses that are widespread in Europe and North America. However, with industrialization and urbanization in the last 20 years, the prevalence of IBD in China has increased, attracting the attention of clinical practitioners and strengthening research into the condition.

IBD is distinguished by hidden asymptomatic intervals and repeated bouts of various degrees of gastrointestinal inflammation (Chang, 2020; Kobayashi et al., 2020; Roda et al., 2020). Blocking the p40 subunit shared by IL-12 and IL-23 was shown to induce colitis, leading to the conclusion that both the IL-12/Th1 and IL-23/Th17 axis may be implicated in the pathophysiology of CD and UC. (Gulati and Dubinsky, 2009; Strober and Fuss, 2011; Chang, 2020).

The considerable decrease in IL-25 in both inflamed intestinal mucosa and serum in patients with dilated IBD and healthy controls, as well as non-inflamed tissues and serum in patients with quiescent UC and CD, is cause for concern. When IBD was treated with infliximab, a TNF-α inhibitor, serum IL-25 levels returned to normal (Su et al., 2013). IL-25 may have a role in the etiology of IBD. Because TC acts as the sole generator of IL-25 in the mucosa, increasing IL-25 expression by TCs is a possible treatment strategy for IBD. The particular role of TC and the location of IL-25 expression, however, remain unknown. Yet, this simply suggests a correlation between IBD and the aberrant TC decrease, not a causal link. The findings presented here, that the quantity of TC in IBD may be altered, will provide vital insights into the underlying mechanism of IBD and clinical practice in the future.

### 8.3 Obesity may be associated with low secretion of IL-25 by TC

Obesity is a globally increasing disease that is a risk factor for the development of a variety of ailments, including numerous cardiovascular issues and digestive system changes. In both rats and humans, diet-induced obesity is characterized by chronic low-grade systemic inflammation as well as alterations in gut flora (Lee et al., 2018). Especially, the proportion of TCs to total epithelial cells was not altered, and TC-specific expression of IL-25 and TLSP was reduced (Arora et al., 2021) along with activation of the GABA_A/B_ receptor pathways, which is positively correlated with alterations in the expression of the TC signature genes IL-25 and TSLP (Arora et al., 2021). This may provide solution for obesity by modulating TC secretion of IL-25 and TLSP.

### 8.4 TC-related DCLK1 may involve in alimentary tumor

DCLK1 is recognized as a possible marker since it is over-expressed in a variety of solid malignant tumors and has been associated to malignant biological activity and poor tumor prognosis (Chandrakesan et al., 2014; Ji et al., 2018).

Under normal circumstances, the only source of DCLK1 is TCs. DCLK1 has been detected in cancer stem cells (CSCs) from esophageal, pancreatic, and colon cancers (May et al., 2009; Vega et al., 2012; Weygant et al., 2015; Cao et al., 2020), suggesting that CSCs are derived from malignant TCs. CSCs interact with the immunosuppressive tumor microenvironment (TME) and aid in the activity of stem cells. An increasing body of data suggests that DCLK1^+^ TCs influence the formation and progression of inflammation-related malignancies (May et al., 2009; May et al., 2010; Vega et al., 2012; Weygant et al., 2015). Konishi et al. (2019) discovered in 2019 that TCs can induce Lgr5^+^ stem cells in the gastrointestinal tract, hence hastening cancer growth. Recent studies have further revealed that gastrointestinal TCs can promote hepatocellular carcinoma (HCC) development by secreting IL-25 to activate macrophages in TME (Friedrich et al., 2019). This “long-distance communication channel of the gut-liver axis” adds a new dimension to the study of TC function. Although TC markers can be found in mouse adenomas, they are uncommon in human cancer cell biopsies (Gerbe et al., 2011; Saqui-Salces et al., 2011), implying that animal studies are not yet useful for speculating on the association between human cancer and TC.

DCLK1 is expressed by certain pancreatic acinar and epithelial cells. Acinar-ductal metaplasia in pancreatic acinar cells may lead to cancer; DCLK1^+^ pancreatic epithelial cells are involved in regeneration following injury or inflammation (according to the lineage-tracing experiment); KRAS mutation in DCLK1^+^ pancreatic epithelial cells in pancreatitis may lead to pancreatic cancer (Nakanishi et al., 2013). Notably, utilizing a DCLK1 kinase inhibitor can reduce these DCLK1^+^ cells in the pancreas (Ferguson et al., 2020). These results suggest that DCLK1 may be a potential target for pancreatic cancer in clinical practice (Cao et al., 2020).

TC has been considered as a source of mature cell-derived carcinogenesis, alongside Paneth cells. In one word, DCLK1^+^ TCs (Nakanishi et al., 2013) or IL17RB^+^ TC-like cells (Goto et al., 2019) have been shown to act as stem cells in an intestinal tumor model. Similarly, in the context of further DSS-induced inflammation, Apc deletion in DCLK1^+^ TCs resulted in the development of colon tumors, whereas no DCLK1-expressing cells developed tumors in the steady state. Furthermore, following an acute assault, intestinal TC can act as colon cancer beginning cells (Westphalen et al., 2014). During validation, however, multiple essential pathways may be implicated in limiting TC activity and TC-derived tumor growth. For example, NF-κB signaling activation may be necessary for non-stem cell dedifferentiation and tumor development. At present, there are still many mysteries in this field. Future research will need to address this issue (Schwitalla et al., 2013).

## 9 Conclusion

We focused on the characteristics and functions of this peculiar cell lineage in this review. TCs secrete various molecules, suggesting that TCs may be associated with intrinsic immunity, intestinal secretion, contraction, pain, fatty acid metabolism, etc. TCs have chemosensory capabilities since they are comparable to tongue taste bud cells. It is worth noting that TC contains SUCNR1, which may detect pathogen invasion. The tuft-ILC2 circuit promotes TC and GC proliferation in type 2 immunity, ultimately expelling pathogens (especially helminth), which is of social significance. To eliminate bacterial infection, TCs also participate in (GPCR-PLCγ2)-Ca^2+^ signaling axis. Furhtermore, TCs might be involved in the gut-brain axis, as well as satiety and energy metabolism.

Diseases associated with TCs are of great concern. Murine TC has been identified as a MNV target. Reduced levels of TC-secreted IL-25 may be linked to IBD, obesity, duodenal ulcer, and acute duodenitis. However, as current studies are still inadequate, there is no more evidence supporting the precise involvement of TCs in these disorders, which is a limitation of our review. Despite the drawbacks, we believe that this evaluation will be useful for future TC-related research. Future research will reveal innovative paths for the diagnosis and treatment of these diseases if the causal link between TCs and the disorders is clarified. DCLK1^+^ cells have been shown to induce tumor growth in the GI tract. Given that TC is the only source of intestinal DCLK1 in the physiological state in mice, it can be hypothesized that carcinogenesis is associated with aberrant TC proliferation. Once the aforesaid molecular mechanisms are elucidated, new approaches for early molecular screening and therapy of GI cancers will emerge.

## Boxes

BOX 1 **Post-mitotic cells**

According to research, TCs are short-lived post-mitotic cells with a lifespan of at least seven days and are regularly replenished (Gerbe et al., 2011). In the “transit-amplifying” zone, the Lgr5^+^ stem cell transformed into shorter-lived cells. Cells continue to move but cease proliferating when they reach the crypt-villus border, resulting in a villus composed entirely of post-mitotic cells.

BOX 2 **Different terminology for cells in the differentiation process and their correspondence**

Bjerknes *et al.* define TA cells as “Mix”, so the “daughters of Mix” was abbreviated as “DOM”, which equals the “daughters of TA cells”. Bjerknes *et al.* refered to DOM entering different states as DOM_Notch_ (Absoprive progenitor) and DOM_Δ_ (Secretory progenitor), respectively.

BOX 3 **TRPM5**

TRPM5 is a critical component of taste transduction, such as bitter, sweet, and umami. It also has a possible role in fat taste signaling (Liu et al., 2011; Mattes, 2011). Expressed in pancreatic β-cells (Colsoul et al., 2010), TRPM5 was proposed to be related to insulin secretion and lower risk of type 2 diabetes in mice (Philippaert et al., 2017). TRPM5 is expressed in sensory cells, including solitary chemosensory cells and TCs.

BOX 4 **Functions of TCs in other organs or tissues**

This cell lineage behaves differently in various organs and tissues than in the gastrointestinal tract. Pancreatic TCs could reduce carcinogenesis by secreting prostaglandins (Delgiorno et al., 2014); tracheal TCs could participate in mucociliary clearance (Perniss et al., 2020); and thymic TCs could take part in the nurture of B cells, NK cells, and T cells (Bornstein et al., 2018).

## Glossary

TC: Tuft cell
GC: Goblet cell
RSEM: Reflection scanning electron microscopy
TEM: Transmission electron microscopy
SEM: Scanning Electron Microscopy
SBF: Serial Block-Face
ATUM: Automated Tape-collecting Ultra-Microtome
IEC: Intestinal epithelial cell
Lgr5: Leucine rich repeat containing G protein-coupled receptor 5
HES1: Hes family bHLH transcription factor 1
ATOH1: Atonal bHLH transcription factor 1
CBC: Crypt basal columnar
ISCs: Intestinal stem cells
IEC: Intestinal epithelial cell
TA cells: Transit amplifying cells (Mix)
M cell: membranous cell
Dll1: Delta like canonical Notch ligand 1
Dll4: Delta like canonical Notch ligand 4
ErbB: Erb-b2 receptor tyrosine kinase
EGF: Epidermal growth factor
BMP: Bone morphogenetic protein
Tcf4: Transcription factor 4
Hes: Hes family bHLH transcription factor
Gfi1b: Growth factor independent 1B transcriptional repressor
Atoh1: Atonal homologue 1 gene, also known as Math1
ADAMs: a Disintegrin and Metalloproteinases
ADAM10: ADAM metallopeptidase domain 10
Spdef: SAM pointed domain containing Ets transcription factor
Klf4: Kruppel-like factor 4
Sox9: SRY-box containing gene 9
Neurog3: Neurogenin 3
Gfi1: Growth factor-independent 1
TRPM5: Transient receptor potential cation channel, and subfamily M, member 5
COX1: Cyclooxygenase 1
COX2: Cyclooxygenase 2
DCLK1: Doublecortin like kinase 1
SUCNR1: Succinate receptor 1
HPGDS: Hematopoietic prostaglandin-D synthase
5HT: Serotonin
IL-25: Interleukin 25
TSLP: thymic stromal lymphopoietin
NO: Nitric oxide
GNAT3: Guanine nucleotide binding protein alpha transducing 3/α-gustducin
ILC2: Group 2 innate lymphoid cell
HpARI: H. polygyrus alarmin released inhibitor
IL-33: Interleukin 33
cysLTs: cysteinyl leukotrienes
IL-4: Interleukin 4
IL-13: Interleukin 13
ACh: Acetylcholine
GPCR: G protein-coupled receptor
PLC: Phospholipase C
PGD2: prostaglandin D2
MNV: murine norovirus
CD300lf: CD300 molecule like family member 1
UC: Ulcerative colitis
CD: Crohn’s disease
PRR: Pattern recognition receptors
TNF-α: Tumor necrosis factor-α
GABA: γ-aminobutyric acid
IL17RB: Interleukin 17 receptor B
NF-κB: Nuclear factor kappa B subunit 1
CSCs: cancer stem cells
IBD: inflammatory bowel disease
GI: Gastrointestinal
DOM: Daughters of TA cells/daughters of Mix
DOM_Notch_: Absoprive progenitor
DOM_Δ_: Secretory progenitor
NK cells: Natural killer cells
HCC: Hepatocellular carcinoma
